# Diagnosis and management of splenic tuberculosis: a case report and literature review

**DOI:** 10.3389/fmed.2025.1622794

**Published:** 2025-09-30

**Authors:** Peijun Liu, Na Liu, Fanghao Li

**Affiliations:** ^1^Department of Respiratory and Critical Care Medicine, The Central Hospital of Enshi Tujia and Miao Autonomous Prefecture, Enshi City, China; ^2^Southern Medical University, Guangzhou, China

**Keywords:** splenic tuberculosis, extrapulmonary tuberculosis, granuloma, histopathology, case report

## Abstract

Splenic tuberculosis is a rare form of extrapulmonary tuberculosis, that is often caused by hematogenous dissemination. A 53-year-old female presented with multiple enlarged lymph nodes and a splenic mass identified during a routine health check. Imaging revealed widespread lymphadenopathy and multiple splenic nodules, raising suspicion for lymphoma. The laboratory findings showed an elevated erythrocyte sedimentation rate and mild liver function abnormalities, with no significant tumor markers or inflammatory indicators. Histopathology of lymph node and splenic biopsies revealed granulomas with epithelioid cells and multinucleated giant cells. Acid-fast staining confirmed the presence of *Mycobacterium tuberculosis*, indicating the presence of splenic tuberculosis. The patient was treated with a standard anti-tuberculosis regimen and adjunctive hepatoprotective therapy, resulting in clinical improvement, and was discharged in stable condition. This case underscores the diagnostic challenges of splenic tuberculosis due to its non-specific presentation and highlights the importance of histopathological evaluation for timely diagnosis and treatment.

## Case presentation

**FIGURE 1:**
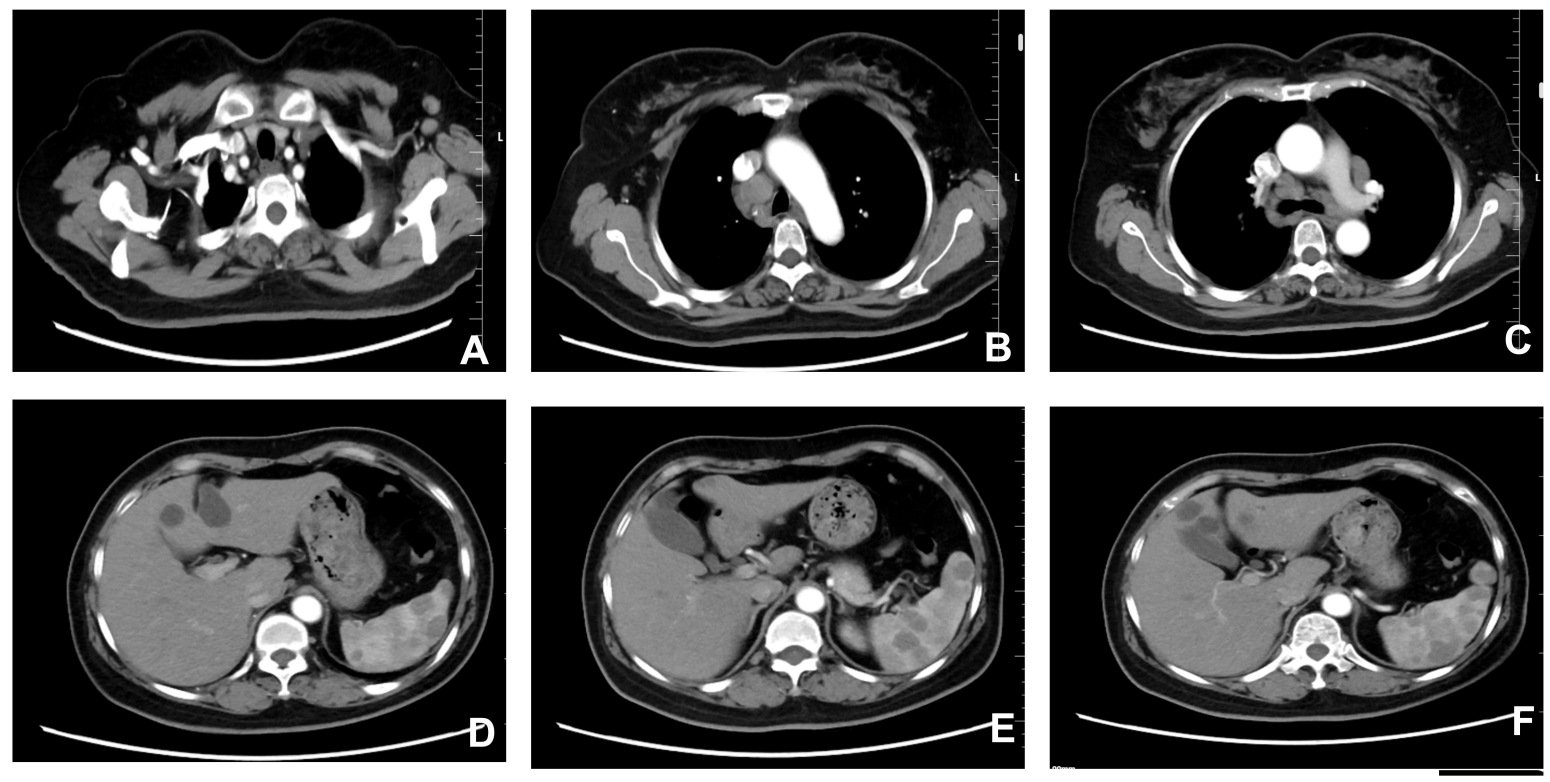
Chest and upper abdominal CT. **(A)** Thoracic inlet level. **(B)** Aortic arch level. **(C)** Carina level. **(D)** Gastric body level. **(E)** Splenic artery level. **(F)** Splenic level. Scale bar = 2 cm.

A 53-year-old female farmer presented at our hospital on September 25, 2024, with a primary complaint of “multiple enlarged lymph nodes and a splenic mass detected during a routine health check-up over the past 20 days.” Enhanced chest and upper abdominal computed tomography demonstrated multiple enlarged lymph nodes involving the mediastinum, bilateral axillary regions, cardiophrenic angles, hepatic hilum, and gastrohepatic ligament, which together raised a strong suspicion for lymphoma. In addition, several hypodense splenic nodules were identified, ranging from a few millimeters to approximately 1 cm in diameter, relatively well-defined, and showing no obvious post-contrast enhancement, features suggestive of diffuse splenic involvement. Pulmonary CT further revealed uneven parenchymal transparency accompanied by fibrous streaks in the right middle lobe, findings consistent with small airway disease and chronic inflammatory changes. Moreover, a small nodule was observed in the upper outer quadrant of the left breast, requiring further evaluation to exclude malignancy. Abdominal imaging also demonstrated mild fatty infiltration of the liver, multiple hepatic nodules radiologically consistent with cysts, and slight thickening at the left adrenal junction, providing a more comprehensive assessment of thoracoabdominal involvement (Figure 1).

## Physical examination upon admission

Upon admission, the patient’s vital signs were as follows: body temperature of 36.7 °C, pulse rate of 85 beats per minute, respiratory rate of 16 breaths per minute, and blood pressure of 150/90 mmHg (1 mmHg = 0.133 kPa). The patient was alert and oriented. Examination of the chest revealed normal bilateral breath sounds with no audible dry or moist rales. Cardiac auscultation revealed a regular heart rhythm with no significant murmurs detected in any valve area. The abdominal examination revealed a soft wall with no tenderness, rebound tenderness, or palpable masses. The liver was not palpable below the costal margin, and there was no tenderness on percussion in the hepatic region. The spleen was not palpable below the costal margin. Both kidneys were free of tenderness on percussion, and no shifting dullness was noted. The extremities were examined, revealing normal range of motion in all limbs. However, bilateral lower limb edema was observed. Neurological examination indicated intact physiological reflexes, with no pathological reflexes elicited. Overall, the clinical examination did not reveal any acute abnormalities requiring immediate intervention, and the findings were consistent with the patient’s stable condition upon admission.

## Laboratory and diagnostic findings

On September 25, 2024, arterial blood gas analysis without supplemental oxygen revealed a pH of 7.44, a partial pressure of carbon dioxide (PCO_2_) of 45.2 mmHg, and a partial pressure of oxygen (PO_2_) of 66.4 mmHg. A complete blood count revealed the decreased white blood cell (WBC) count of 3.50 × 10^9^/L, an elevated neutrophil percentage of 72.60%, and an absolute neutrophil count of 2.54 × 10^9^/L. Red blood cell count (RBC) was 4.26 × 10^12^/L, hemoglobin (Hb) was 124 g/L, and platelet count was 173 × 10^9^/L. Erythrocyte sedimentation rate (ESR) was elevated at 56.0 mm/hr. Liver function tests revealed mildly elevated alkaline phosphatase (ALP) at 99 U/L and gamma-glutamyl transferase (GGT) at 88 U/L, alongside a serum uric acid level of 393.12 μmol/L. Other tests, including cardiac enzyme profiles, procalcitonin (PCT), carcinoembryonic antigen (CEA), disseminated intravascular coagulation (DIC) panel, stool routine, urine routine, and C-reactive protein (CRP), were within normal limits.

**FIGURE 2:**
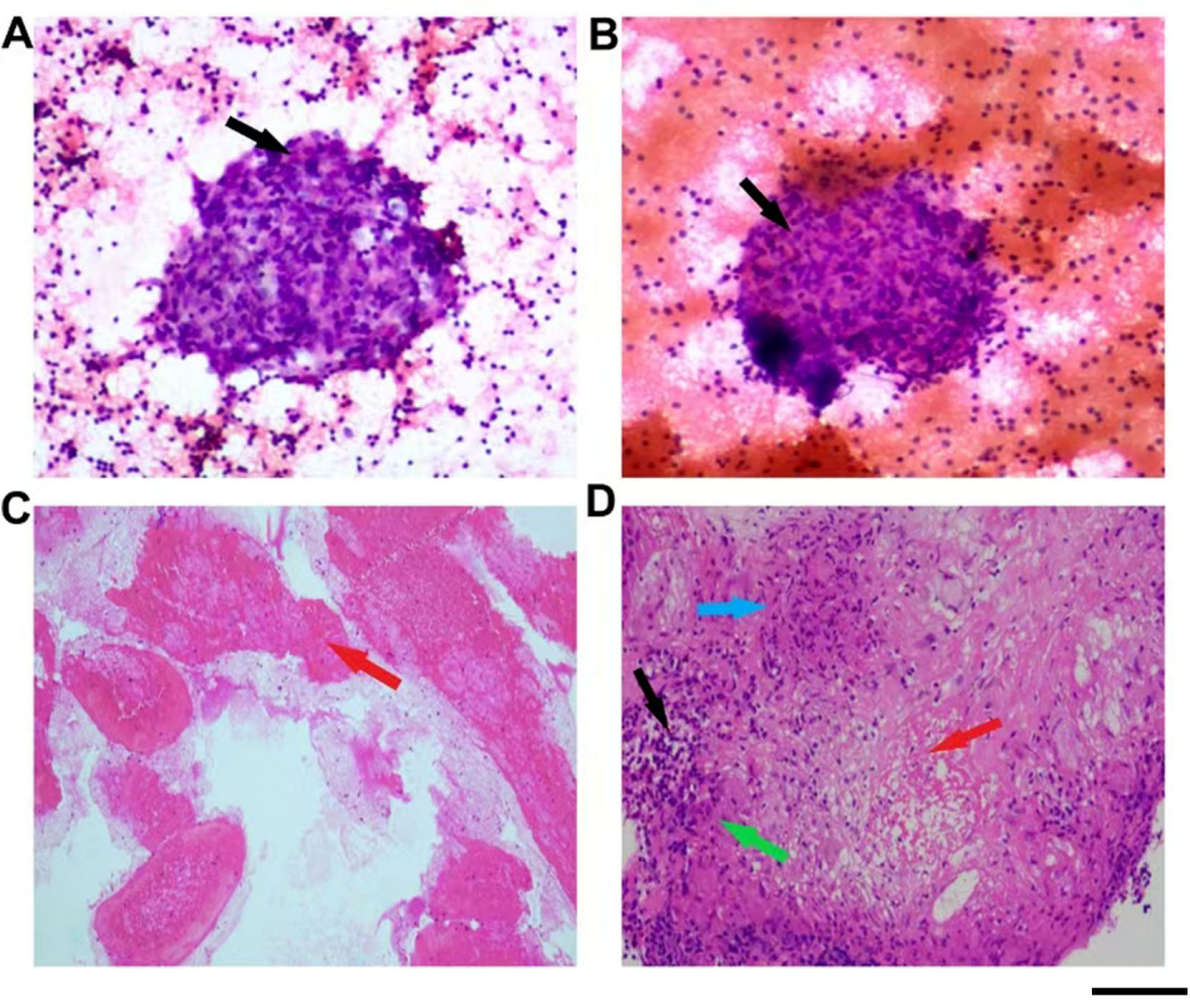
Pathological examination findings. **(A)** Pathological features of the axillary lymph node fine needle aspiration. **(B)** Pathological features of the cervical lymph node fine needle aspiration. **(C)** Pathological features of pulmonary lymph node fine needle aspiration. **(D)** Pathological features of the splenic fine needle aspiration. The black arrow indicates mononuclear macrophages, the red arrow indicates necrotic tissue, the green arrow indicates epithelioid cells, and the blue arrow indicates multinucleated giant cells. Scale bar = 100 μm.

Imaging studies and further diagnostic procedures provided critical insights. Echocardiography indicated a thickened interventricular septum, left atrial enlargement, and impaired left ventricular diastolic function. Ultrasound-guided fine needle aspiration biopsy of the left axillary and cervical lymph nodes revealed numerous epithelioid cells arranged in a sheet-like pattern within a lymphocytic background, with occasional multinucleated giant cells, which was consistent with chronic granulomatous lymphadenitis (Figures 2A, B). Bronchoscopy with acid-fast and fungal staining of bronchial brushings showed no abnormalities. Endobronchial ultrasound-guided transbronchial needle aspiration (EBUS-TBNA) of 4R lymph nodes demonstrated blood clots and a small number of distorted lymphocytes in histological and cytological smears. Cytological examination of lavage fluid also revealed few scant distorted lymphocytes (Figure 2C).

The definitive diagnosis was established through splenic lesion biopsy, which revealed fibrotic tissue containing granulomas formed by epithelioid cells and multinucleated giant cells, accompanied by lymphocytic infiltration. Acid-fast staining identified acid-fast bacilli within the splenic tissue, confirming tuberculosis (Figure 2D).

## Treatment and outcome

The patient was diagnosed with splenic tuberculosis accompanied by granulomatous lymphadenitis. Anti-tuberculosis therapy was initiated with a standard regimen consisting of an intensive phase of 2 months with isoniazid, rifampin, pyrazinamide, and ethambutol (HRZE), followed by a continuation phase of 14 months with isoniazid and rifampin (HR). The total daily oral dose was 3600 mg, administered together with hepatoprotective and symptomatic treatments. Following treatment, the patient showed significant clinical improvement and was discharged in stable condition. On subsequent follow-up, the patient remained asymptomatic after completion of anti-tuberculosis therapy and did not experience any treatment-related adverse effects. She continues to be monitored under long-term clinical follow-up, with no evidence of recurrence observed to date. The diagnostic and therapeutic progression is illustrated in the flow diagram shown in Supplementary Figure 1.

## Discussion

Upon admission, the patient presented with multiple enlarged lymph nodes and a splenic mass. The involvement of the mediastinal, bilateral axillary, cardiophrenic, hepatic hilum, and gastrohepatic lymph nodes indicated extensive lymphatic system involvement. Imaging findings of multiple splenic nodules suggested a strong association with lymphatic system diseases. These clinical features raised the following differential diagnoses.

### Malignant lymphoma

Lymphoma typically manifests as painless, progressive lymphadenopathy with or without systemic symptoms such as fever, night sweats, and weight loss. As a heterogeneous group of malignancies comprising over 90 subtypes of lymphoid neoplasms, lymphoma accounts for approximately 82,000 new cases annually in the United States. Risk factors include smoking, obesity, genetic predisposition, infections, and inflammatory responses (1). In contrast to typical cases of diffuse large B-cell lymphoma, our case of splenic tuberculosis is exceedingly rare, with non-specific clinical features and imaging findings of a large splenic mass that can easily mimic malignant lymphoma (2). However, in this case, pathological examination revealed no evidence of malignant lymphoid infiltration, ruling out a diagnosis of lymphoma.

### Non-infectious granulomatous diseases

These include conditions such as sarcoidosis and immune-related disorders (e.g., rheumatic diseases). Non-infectious granulomatous diseases are characterized by granuloma formation and are not caused by conventional pathogens such as bacteria, viruses, fungi, or parasites, but rather by aberrant immune responses (3). Representative conditions include sarcoidosis and certain immune-mediated diseases. Sarcoidosis is a systemic granulomatous disorder of unknown etiology that can affect multiple organ systems, most commonly the lungs and lymphatic system. It is characterized by the formation of non-caseating granulomas, typically without significant necrosis. While the exact cause of sarcoidosis remains unclear, it is thought to involve a complex interplay of genetic, environmental, and immune system factors. Clinically, sarcoidosis may present with symptoms such as cough, dyspnea, fatigue, and fever, and in severe cases, it can involve the heart, eyes, skin, and other organs. Treatment primarily relies on corticosteroids and other immunosuppressants (4, 5). Immune-related diseases encompass a wide range of conditions associated with immune system dysregulation, such as rheumatic diseases, systemic lupus erythematosus, and rheumatoid arthritis. These conditions are typically characterized by chronic inflammation that can affect multiple organ systems, including joints, skin, and kidneys. The pathogenesis of immune-related diseases is complex, involving a combination of autoimmune responses, genetic predisposition, and environmental triggers (6, 7). However, in this case, the absence of typical systemic manifestations, such as pulmonary reticular opacities and elevated antinuclear antibody levels, makes these conditions less likely. Further investigations led to the identification of acid-fast bacilli in the splenic biopsy, confirming tuberculosis as the underlying cause. This highlights the importance of a thorough diagnostic workup in cases with overlapping clinical features, ensuring accurate differentiation between infectious, malignant, and immune-related conditions.

### Infectious diseases

Infectious diseases, including tuberculosis, fungal infections, or other pathogen-induced conditions, can result in lymph node and splenic abnormalities. These diseases are caused by a wide range of pathogens, including bacteria, viruses, fungi, and parasites (8). When the human body is invaded by these pathogens, the immune system initiates an inflammatory response to combat the infection, often leading to pathological changes in immune organs such as the lymph nodes and spleen (9). Tuberculosis (TB), caused by *Mycobacterium tuberculosis*, is a chronic infectious disease primarily affecting the lungs but capable of spreading to other parts of the body, including the lymph nodes and spleen (10). Approximately 10 million people are infected with TB annually worldwide, with 75% involving pulmonary disease. Prompt diagnosis and targeted treatment are critical for controlling disease progression and transmission. However, for latent TB infection, the absence of specific biomarkers and limited clinical trial data make diagnosis and treatment decisions challenging (11). During the progression of TB, the pathogen induces characteristic pathological changes at the infection site, most notably granuloma formation. Granulomas represent the host’s immune response to persistent antigenic stimulation (12, 13). These structures are composed of aggregated tissue cells, often including epithelioid cells and multinucleated giant cells, known as Langhans giant cells, which form through macrophage fusion as part of the immune defense against infection (14). Studies have demonstrated that transplanting Langhans giant cells into a murine skin TB infection model significantly reduced bacterial load, suppressed granuloma growth, and enhanced the expression of antimicrobial peptides and inflammatory cytokines (15). Recent advances in high-dimensional imaging using MIBI-TOF technology have provided detailed insights into the immune microenvironment of active TB granulomas. This technique has enabled the construction of a TB granuloma atlas, identifying 19 cellular subpopulations and 8 spatial microenvironments. These findings highlight the critical roles of IFN-γ deficiency, TGF-β enrichment, and immune regulatory cells within TB granulomas. Additionally, peripheral blood transcriptomic analyses have shown that immune regulatory trends align with granuloma characteristics, with PD-L1 expression associated with TB progression and treatment response. These results underscore the importance of local immune regulation in mediating systemic effects of TB (16).

### Splenic tuberculosis

Splenic tuberculosis is a relatively rare form of tuberculosis, typically caused by hematogenous dissemination. Unlike pulmonary tuberculosis, splenic tuberculosis presents with non-specific symptoms, which may include abdominal discomfort, pain, generalized fatigue, fever, night sweats, and weight loss. In some cases, it may manifest as an atypical asymptomatic condition in patients without underlying diseases (17). Owing to the spleen’s concealed anatomical location, its symptoms are often overlooked or mistaken for other abdominal conditions, leading to delayed diagnosis. Studies analyzing splenic tuberculosis in patients undergoing non-traumatic splenectomy have shown that it can present with unexplained fever and splenomegaly, often accompanied by hepatomegaly or other tuberculosis-related lesions. However, in some cases, it may occur as isolated splenic tuberculosis (18). Splenic tuberculosis can also occur in immunocompetent individuals, often with atypical imaging and clinical features, consistent with the presentation in this case. Compared with diffuse large B-cell lymphoma, which typically presents with well-defined pathological and immunohistochemical features, our case of splenic tuberculosis is exceptionally rare and can easily mimic malignant lymphoma in clinical and imaging findings. Its novelty lies in the confirmation of an infectious lesion rather than a neoplasm, underscoring the importance of thorough differential diagnosis (19). In contrast, pulmonary tuberculosis typically exhibits more pronounced symptoms, including chronic cough, hemoptysis, chest pain, fever, night sweats, and weight loss. Diagnosis primarily relies on chest X-rays, CT scans, and sputum acid-fast staining and culture (20). Research indicates that chest CT is more accurate than chest X-rays in diagnosing tuberculosis in children. In cases of suspected pulmonary tuberculosis, CT detection rates (70.3%) are significantly higher than those of chest X-rays (36.6%) (21). In terms of treatment, the standard regimen for pulmonary tuberculosis involves a 6-month course of multiple anti-tuberculosis drugs, including isoniazid, rifampin, pyrazinamide, and ethambutol (22). Although the treatment principles for splenic tuberculosis are similar, it often requires close monitoring for disease progression due to the potential for more extensive dissemination. Adjustments to drug regimens and treatment duration may be necessary. In certain cases, if medical therapy proves ineffective or severe complications arise, surgical removal of the affected spleen may be required. Owing to resource limitations, cytokine profiling was not conducted in this case. We recognize this as a limitation and recommend that future studies incorporate cytokine profiling to further elucidate the immunopathological mechanisms of splenic tuberculosis.

## Conclusion

Tuberculosis remains a major global health challenge, with its incidence influenced by socioeconomic conditions, comorbidities, and healthcare disparities. Splenic tuberculosis, though rare, is an important manifestation of extrapulmonary disease. Histopathology provides supportive evidence through granulomatous features, while microbiological confirmation such as acid-fast staining is valuable whenever obtainable, although the confirmatory diagnosis of tuberculosis can only be established by culture. Our case demonstrated acid-fast bacilli together with typical histopathological changes, supporting the diagnosis. Standard anti-tuberculosis therapy remains the cornerstone of management, with surgery reserved for complicated situations. As splenic tuberculosis may occur as part of disseminated disease, comprehensive systemic evaluation and timely intervention are essential for optimal outcomes.

## Data Availability

The original contributions presented in this study are included in this article/Supplementary material, further inquiries can be directed to the corresponding author.

## Supplementary material

Supplementary Figure 1 Diagnostic and therapeutic progression of the patient with splenic tuberculosis.
