# Supplementary figures and images for: Diagnosis and management of splenic tuberculosis: a case report and literature review
Source: Front Med (Lausanne). 2025 Sep 30;12:1622794. doi: 10.3389/fmed.2025.1622794 (PMC12519452; doi:10.3389/fmed.2025.1622794)

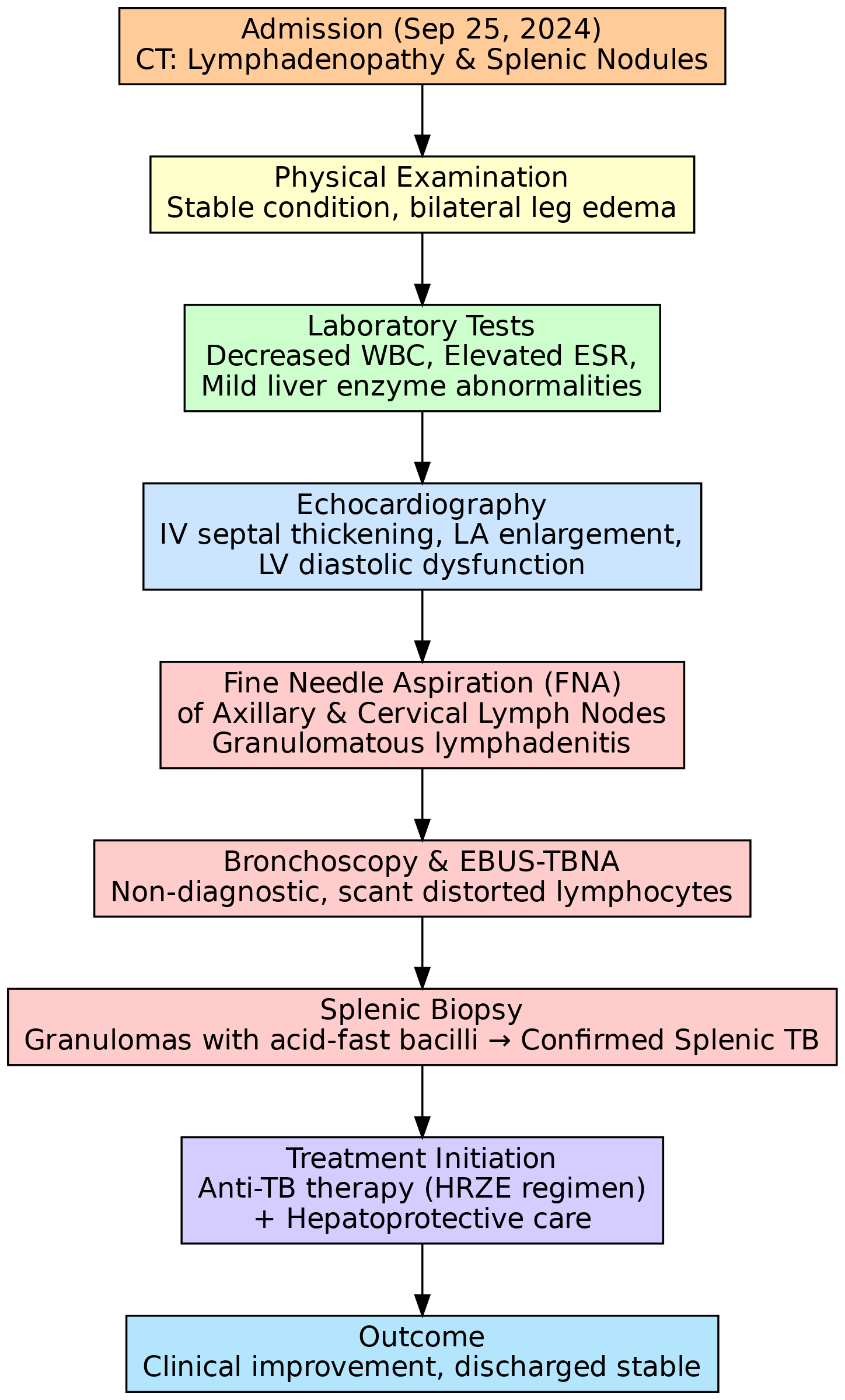

Supplement: Supplementary Figure 1 — Diagnostic and therapeutic progression of the patient with splenic tuberculosis. [file Image_1.tif]
